# Cross-institutional evaluation of deep learning and radiomics models in predicting microvascular invasion in hepatocellular carcinoma: validity, robustness, and ultrasound modality efficacy comparison

**DOI:** 10.1186/s40644-024-00790-9

**Published:** 2024-10-22

**Authors:** Weibin Zhang, Qihui Guo, Yuli Zhu, Meng Wang, Tong Zhang, Guangwen Cheng, Qi Zhang, Hong Ding

**Affiliations:** 1 Department of Ultrasound, Huashan Hospital, Fudan University, Shanghai, 200040 The People’s Republic of China; 2 The SMART (Smart Medicine and AI-based Radiology Technology) Lab, School of Communication and Information Engineering, Shanghai University, Shanghai, 200444 The People’s Republic of China; 3 Department of Ultrasound, Institute of Ultrasound in Medicine and Engineering, Zhongshan Hospital, Fudan University, Shanghai, 200032 The People’s Republic of China; 4 National Clinical Research Center for Aging and Medicine, Huashan Hospital, Fudan University, Shanghai, The People’s Republic of China

**Keywords:** Deep learning, Radiomics, Contrast-enhanced ultrasound, Microvascular invasion, Head-to-head comparison

## Abstract

**Purpose:**

To conduct a head-to-head comparison between deep learning (DL) and radiomics models across institutions for predicting microvascular invasion (MVI) in hepatocellular carcinoma (HCC) and to investigate the model robustness and generalizability through rigorous internal and external validation.

**Methods:**

This retrospective study included 2304 preoperative images of 576 HCC lesions from two centers, with MVI status determined by postoperative histopathology. We developed DL and radiomics models for predicting the presence of MVI using B-mode ultrasound, contrast-enhanced ultrasound (CEUS) at the arterial, portal, and delayed phases, and a combined modality (B + CEUS). For radiomics, we constructed models with enlarged vs. original regions of interest (ROIs). A cross-validation approach was performed by training models on one center’s dataset and validating the other, and vice versa. This allowed assessment of the validity of different ultrasound modalities and the cross-center robustness of the models. The optimal model combined with alpha-fetoprotein (AFP) was also validated. The head-to-head comparison was based on the area under the receiver operating characteristic curve (AUC).

**Results:**

Thirteen DL models and 25 radiomics models using different ultrasound modalities were constructed and compared. B + CEUS was the optimal modality for both DL and radiomics models. The DL model achieved AUCs of 0.802–0.818 internally and 0.667–0.688 externally across the two centers, whereas radiomics achieved AUCs of 0.749–0.869 internally and 0.646–0.697 externally. The radiomics models showed overall improvement with enlarged ROIs (*P* < 0.05 for both CEUS and B + CEUS modalities). The DL models showed good cross-institutional robustness (*P >* 0.05 for all modalities, 1.6–2.1% differences in AUC for the optimal modality), whereas the radiomics models had relatively limited robustness across the two centers (12% drop-off in AUC for the optimal modality). Adding AFP improved the DL models (*P* < 0.05 externally) and well maintained the robustness, but did not benefit the radiomics model (*P >* 0.05).

**Conclusion:**

Cross-institutional validation indicated that DL demonstrated better robustness than radiomics for preoperative MVI prediction in patients with HCC, representing a promising solution to non-standardized ultrasound examination procedures.

## Introduction

Hepatocellular carcinoma (HCC) is the fourth most common cause of cancer-related deaths worldwide [1]. Imaging examinations play an important role in the diagnosis and treatment of HCC, providing preoperative qualitative, localization, and lesion size assessments. However, for patients with resectable HCC assessed before surgery, the postoperative recurrence rate remains high (50–70% in 5 years after surgery), resulting in a large consumption of medical resources and contributing to an increase in mortality [2]. Therefore, if more accurate histological information can be provided through preoperative imaging examinations, it is expected to help individualize treatment for patients with different risk levels and reduce the risk of postoperative recurrence in patients with HCC.

Currently, the clinical diagnosis of microvascular invasion (MVI) status can only be determined by postoperative pathology. The feasibility of preoperative morphological assessment of MVI has been explored. A meta-analysis on magnetic resonance imaging (MRI) and computed tomography (CT) assessment of MVI showed that morphological assessment achieved an area under the curve (AUC) ranging from 0.62 to 0.72 [3]. However, significant imaging findings are limited on CT or MRI and are still debated, mainly due to the lack of imaging features [4–7]. Quantitative methods, such as radiological nomogram, diffusion kurtosis imaging (DKI) of MRI, or time-intensity curve (TIC) of contrast-enhanced ultrasound (CEUS), have moderate diagnostic efficacy [8–12], where post-processing is more complex than conventional imaging and some methods still carry certain subjective factors in the process. For the preoperative assessment of histological indices, it is difficult to accurately diagnose morphological findings or quantitative indices.

Deep learning (DL) and radiomics have become increasingly popular in medical imaging. Several studies have sought to develop radiomics models based on CT/MRI for preoperative estimation of MVI in patients with HCC, with an AUC reported in the range of 0.744 to 0.942 [9, 13, 14]. Although determining MVI status via imaging examination before surgery is challenging, the use of radiomics and DL has significantly improved diagnostic accuracy. Numerous studies have proposed radiomics and DL models for MVI prediction. However, the most efficient modeling methodology, cross-center robustness, and optimal image modality remain debated given the varying performance across different methods and centers.

CEUS, as a real-time and operator-dependent technique, faces challenges in standardized imaging, which differs from the standardized imaging protocols of MRI and CT. To achieve model performance comparable to that of MRI and CT-based methods, it is necessary to address the standardization issues in CEUS imaging. In this study, we aimed to analyze the efficacy of DL and radiomics models in predicting MVI in HCC using CEUS images. We aimed to overcome the standardization challenges of CEUS and develop robust, generalizable models for MVI prediction in patients with HCC that are comparable to enhanced MRI or CT models. The findings of this study could enhance the clinical utility of CEUS in preoperative MVI assessment, contributing to improved patient management and treatment planning. To the best of our knowledge, no previous study has conducted a head-to-head comparison between DL and radiomics models for ultrasound modalities, or investigated model robustness and generalizability through rigorous internal and external validation across centers.

## Materials and methods

### Patients and clinical data

A total of 576 patients were enrolled from two centers: 286 from center 1 (April 2018 to January 2022) and 290 from center 2 (October 2020 to January 2022). Patients were included according to the following criteria: (1) patients with HCC confirmed histopathologically after surgical resection, with tumor estimation including MVI status and liver Scheuer grade and stage in documentation; (2) who underwent conventional ultrasound and CEUS examination before surgery; and (3) patients with multiple lesions had the largest one enrolled.

The exclusion criteria were as follows: (1) patients with complicated clinical conditions, such as pregnancy and taking medication for collagen diseases; and (2) patients who received additional treatment before examination, such as chemotherapy, radiofrequency ablation (RFA), or transcatheter arterial chemoembolization (TACE). Clinical information was collected within 2 weeks before surgery, including age, sex, alpha-fetoprotein (AFP) levels, and serum biomarkers of hepatitis.

### Acquisition of ultrasound images

Conventional and contrast-enhanced ultrasound (Sonovue^®^ by Bracco, Italy) images were retrospectively obtained on iU22, EPIQ7 (Philips, Andover, MA, USA), LOGIQ E9 (GE, London, UK), Aplio 500 (Canon, Tokyo, Japan), and MyLab Twice (Esaote, Milan, Italy) instruments. For each lesion, one still image in B-mode and three still images of CEUS in the arterial phase (AP; 10–30 s), portal phase (PP; 31–120 s), and delayed phase (DP; 121– s), were selected by a senior sonographer with more than 5 years of experience in liver CEUS examination. The criteria for image selection were as follows: (1) images showing lesions with liver parenchyma background and (2) images > 1 cm and < 10 cm. The exclusion criteria for images were as follows: (1) unclear images of lesions or liver parenchyma; (2) the lesion was too deep to exhibit intralesional details; and (3) insufficient examination of the target lesion (or image data missing). A total of 2304 images were obtained from the two centers’ databases.

### Clinical diagnosis of MVI

MVI status was evaluated by two sonographers with over 5 years of experience in CEUS. Based on the recommended criteria from the Surveillance, Epidemiology, and End Results (SEER) database for predicting MVI [15], 5.3 cm in diameter for solitary HCC lesions and 50 mm for multiple lesions on ultrasound were used as the ultrasound consensus criteria for assessing MVI status in our study. Lesions > 5 cm were considered MVI-positive. For lesions ≤ 5 cm, the two sonographers evaluated the tumor margin characteristics, completeness of the capsule, and presence of portal vein invasion to determine the MVI status [3]. Any differences in assessments between the sonographers were resolved through discussion to reach a consensus.

### Histopathological examination of MVI

All hepatic specimens were reviewed by a hepatic pathologist with more than 15 years of experience in hepatic pathology. According to the practice guidelines of the Chinese Society of Pathology, MVI was defined based on the tumor cells (> 50) that could be found in the endothelial vascular lumen under microscopy [16]. MVIs were documented as follows: M0, no MVI; M1 (low-risk group), ≤ 5 MVI in adjacent liver tissue ≤ 1 cm away from the tumor; and M2 (high-risk group), > 5 MVI or MVI in liver tissue > 1 cm away from the tumor. In this study, lesions with M0 were included in the MVI-negative group, and those with M1 or M2 were included in the MVI-positive group.

### Datasets for radiomics and DL models for predicting MVI

**Fig. 1:**
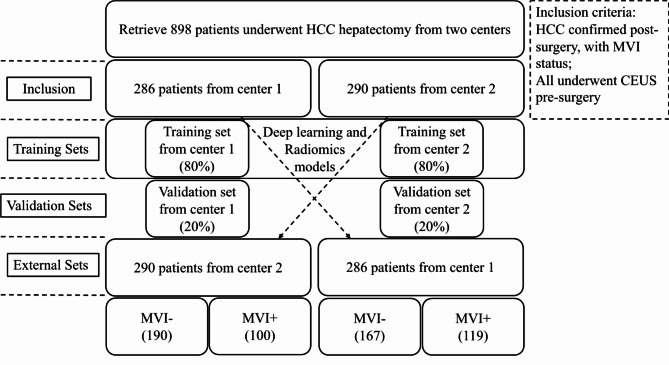
Flowchart of patient enrollment and dataset setup

We collected 576 HCC cases with MVI estimation from two centers. Center 1 contributed 286 HCC cases and center 2 contributed 290 HCC cases. We used data from both centers to construct the radiomics and DL models by training models on data from center 1 and testing models on data from center 2, and vice versa. For model development within each center, an 8:2 random split was used: 80% of the data were used for model training, and 20% were used for internal validation. This allowed for rigorous cross-institutional validation of the models to assess their generalizability and robustness. Figure 1 shows a flowchart of the data setup.

### Sample size calculation

In previous studies, the DL model based on enhanced CT achieved an AUC of 0.75 in predicting MVI in HCC, whereas the machine learning model achieved an AUC of 0.68 [16]. The positive rate of MVI was approximately 45%. Using PASS 2021 (ver. 21.0.3) software for sample size calculation, it was determined that 260 cases were needed for each group (98 MVI positive and 162 negative cases) to detect the AUC difference between the DL and radiomics methods. Considering the need for cross-center validation, the sample size was doubled to approximately 600 cases.

### Model construction using radiomics and DL methods

**Fig. 2:**
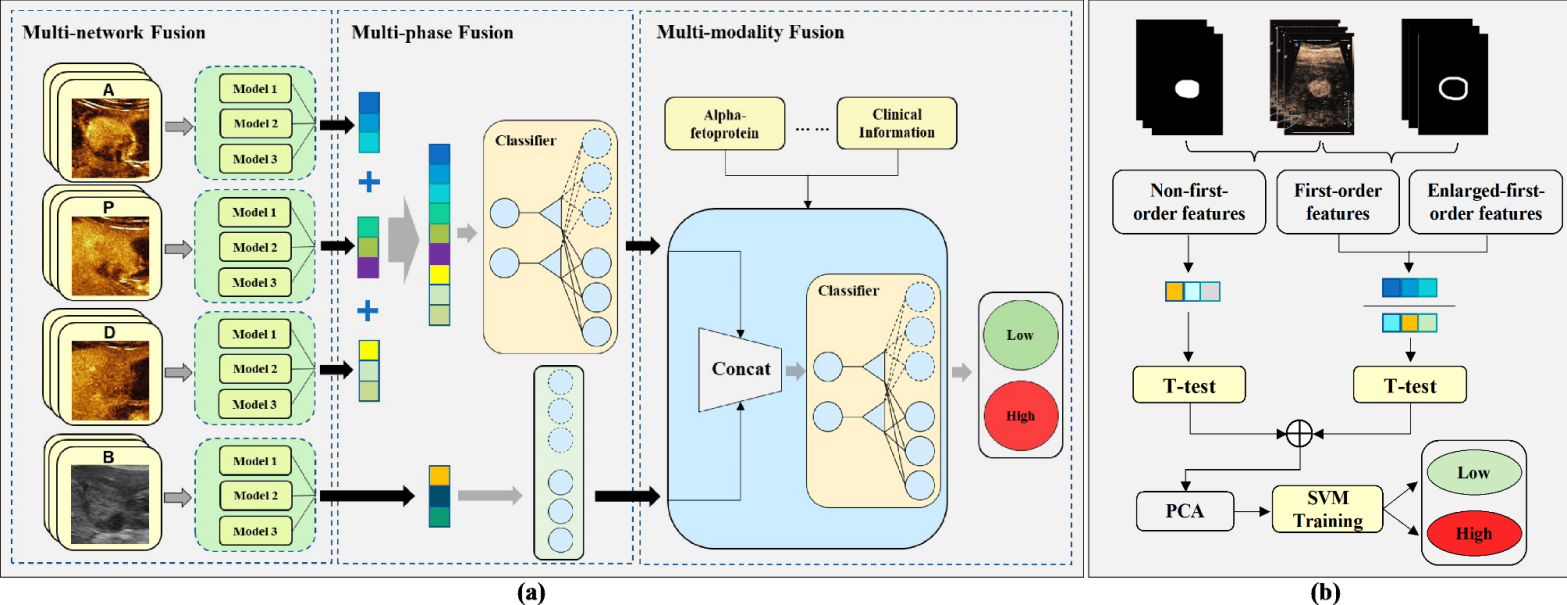
Workflow of DL and radiomics model construction. (**a**) The DL model construction involved multi-network fusion on each modality, multi-phase fusion to integrate CEUS features, modality fusion to incorporate B-mode features, and integration of clinical factors, culminating in a comprehensive prediction, model1: ResNet50, model2: Swin Transformer, model3: CSWin Transformer. (**b**) The radiomics model construction involved ROI expansion, feature extraction on original and expanded ROIs, feature selection by statistical filtering and PCA, and final SVM classifier training

We developed DL and radiomics models using B-mode ultrasound and CEUS at AP, PP, and DPs, and combined modalities (B + CEUS). Figure 2a shows the DL model process. For each image type, we trained three neural networks (ResNet50, Swin Transformer, and CSWin Transformer) and combined their results via ensemble learning (multi-network fusion). ResNet50 is a 50-layer deep convolutional network that uses residual blocks to address vanishing gradients. Swin Transformer uses a hierarchical structure and local window self-attention for large-scale image processing. CSWin Transformer employs cross-window self-attention in the horizontal and vertical directions to realize efficient global feature representation. CEUS AP, PP, and DP images, after multi-network fusion, produced three features each, which were then input into a classifier for ensemble learning (multi-phase fusion). The multi-phase fusion result was combined with the B-mode multi-network fusion result in another classifier that integrated the B-mode and CEUS features. Lastly, we added clinical factors such as AFP to this classifier, thereby creating a comprehensive prediction that combined multi-network, multi-phase, multi-modality, and clinical data for optimal MVI grading prognosis. Additionally, data augmentation were applied in DL method to increase the diversity of the training set, including affine transformations such as rotation, cropping, and flipping to improve the model’s generalizability.

Figure 2b displays the construction process of the radiomics model. For the included images, we extracted 942 features using PyRadiomics (https://www.radiomics.io/pyradiomics). Image selection and segmentation were conducted on a de-identified dataset, which avoided selection bias and ensured patient privacy. Based on the tumor ROI outlined by the sonographers using ITK-SNAP software (http://www.itksnap.org), and we performed automatic morphological expansion of the original ROI by 20% to include information from the surrounding liver parenchyma. We then performed feature selection using a t-test and least absolute shrinkage and selection operator (LASSO), followed by dimensionality reduction using principal component analysis (PCA), retaining dimensions corresponding to 85% of the information content. For cross-institutional validation, when using center 1 for training and validation and center 2 for testing, we extracted the features from center 2 that corresponded to those retained after the t-test and LASSO feature selection in center 1. We then performed PCA dimensionality reduction to ensure consistent features between the two centers. Subsequently, the combined features were trained in an SVM classifier, achieving optimal radiomics model prediction for MVI classification.

### Heatmap

We next conducted heatmap analysis to better understand the decision-making process of our DL models for MVI grading prognosis. Heatmap visualization techniques highlight the image regions that the model focuses most on when making predictions. We performed heatmap analysis separately on the ResNet50, Swin Transformer, and CSWin Transformer networks to compare their focus areas in B-mode and CEUS images (including AP, PP, and DPs). The analysis revealed distinct strengths of the network architectures; ResNet50’s heatmaps concentrated on local details, while Transformer-based models (Swin and CSWin) captured broader contextual information. A comparison of B-mode and CEUS image heatmaps showed effective integration of information from different modalities, especially during multi-phase fusion. This approach provided valuable insights for both clinicians and model architects, enhancing the understanding of the model’s decision-making process and improving its credibility for clinical use. Figure 4 shows one MVI-negative B-mode image and three AP images with different MVI statuses, along with their respective heatmaps.

### Statistics and analysis

The distributions of the clinical characteristics of the patients at the two centers were evaluated using the chi-square test for categorical data and Mann–Whitney U test for continuous data. For model evaluation, the AUC was calculated for all models and the accuracy, sensitivity, specificity, negative predictive value (NPV), and positive predictive value (PPV) were also calculated from the confusion matrix. For DL and radiomics methods, the Delong test was used to assess cross-center robustness between two centers within in/external datasets. For Radiomics method, the Delong test was performed between original and enlarged ROIs within the same modalities. Finally, the Delong test was performed between combined AFP models vs. optimal models.

**Fig. 3:**
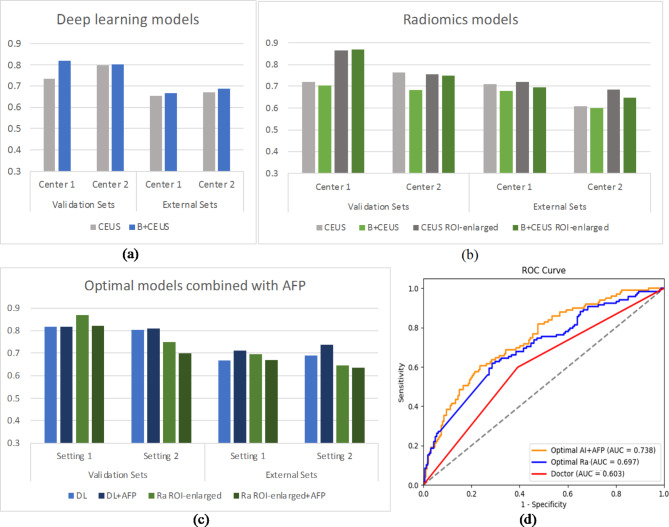
AUC comparison of DL and radiomics models. (**a**) DL models in CEUS and B + CEUS modalities across centers. (**b**) Radiomics in CEUS and B + CEUS modalities with original and enlarged ROIs. (**c**) The optimal DL and radiomics models with clinical factor. (**d**) ROC curves comparing the best models with the doctor

**Fig. 4:**
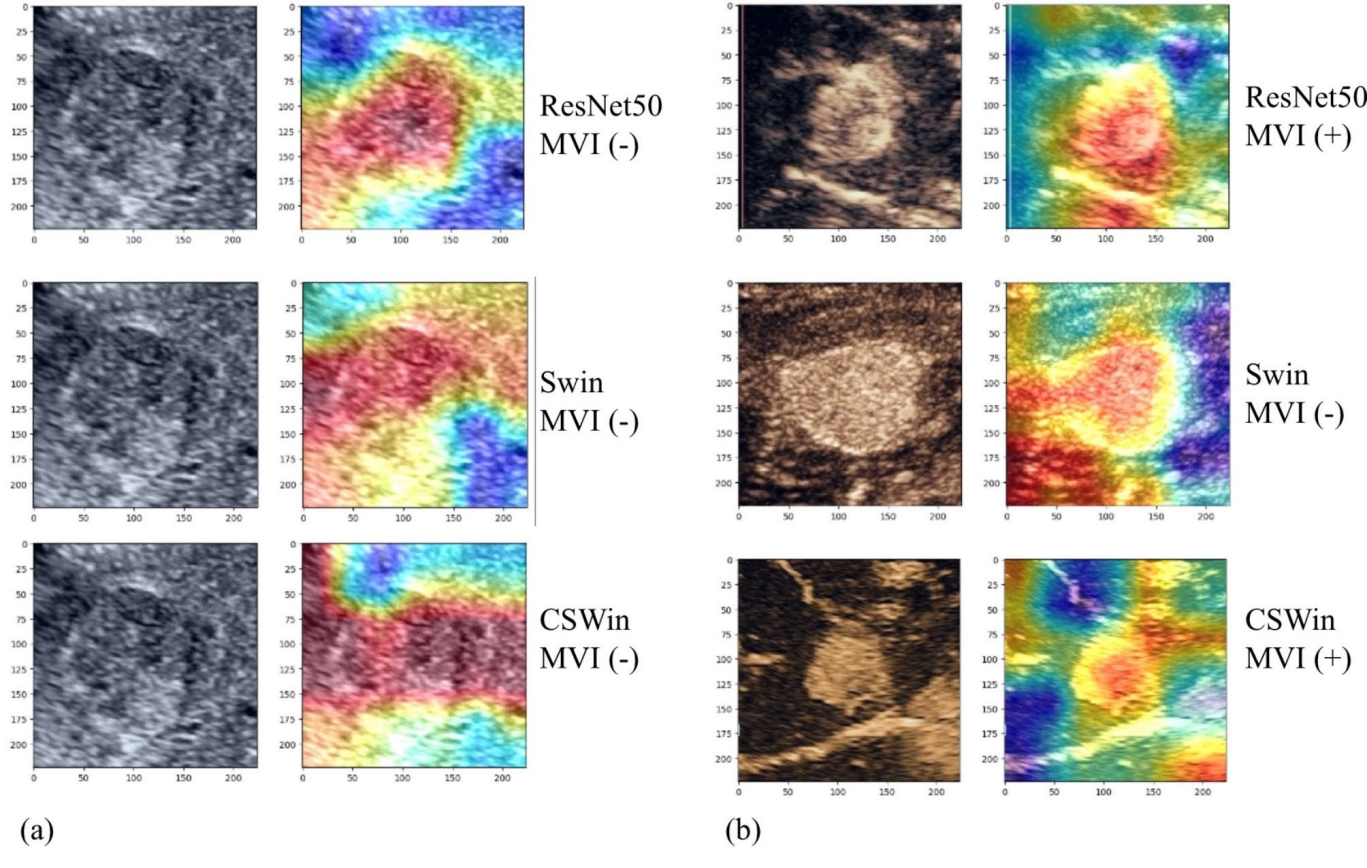
Heatmap: The image demonstrates the decision-making process of DL models in MVI grading prognosis. (**a**) B-mode ultrasound images and their corresponding heatmaps, with ResNet50’s heatmaps focusing on local details, while Transformer-based models (such as Swin and CSWin) capture broader contextual information. (**b**) Three cases of arterial phase images and their respective heatmaps, exhibiting a high degree of matching

## Results

Table 1 presents the baseline characteristics of the two centers and datasets. A total of 119 (41.6%) of the 286 patients in center 1 were pathologically determined to be MVI-positive compared to 100 (52.6%) of the 290 patients in center 2. The patients from each center were randomly distributed into the training and validation sets to maintain a similar MVI distribution.

**Table 1:** Baseline characteristics of the patients in the two centers

Characteristic	Center 1	Center 2	*P*-value
Patient number	286	290	
Age	59.23 ± 10.395	59.74 ± 10.79	0.571
Sex			0.345
Male	242	237	
Female	44	53	
Size (cm)	41.35 ± 26.59	39.83 ± 24.38	0.326
≤ 5	206	215	
> 5	80	75	
HBV	273	270	0.225
HCV	4	8	0.383
AFP (ng/ml)			0.429
< 20	166	168	
20–200	53	59	
> 200	67	63	
MVI positive	119	100	0.078
GS Stage			
≤ G2	268	271	1.095
> G2	18	19	-
≤ S2	98	92	0.610
> S2	188	198	-

AFP: Alpha-fetoprotein, HBV: Hepatitis B virus, HCV: Hepatitis C virus, MVI: Microvascular invasion, GS: Scheuer GS classification, G: Grade of inflammation, S: Stage of fibrosis

### DL and radiomics models for MVI prediction

We applied two model construction strategies to build 12 DL models and 24 radiomics models separately for each ultrasound modality. For DL models, the B + CEUS modality had the highest AUCs of 0.818 and 0.802 within the internal validation sets and 0.667 and 0.688 in the cross-institutional external sets. The Delong test showed no significant difference during the cross-institutional comparison (*P >* 0.05, for all modalities), with differences in AUCs of only 1.6–2.1% for the optimal modality, indicating good cross-institutional robustness of the optimal DL models. The performance of DL models is shown in Table 2.

**Table 2:** Evaluation of DL performance on MVI prediction via reciprocal cross-institutional validation on multidata

DL model AUCs	Validation sets		ΔAUC (%)	External sets		ΔAUC (%)
Modality	Center 1	Center 2		Center 1	Center 2	
B	0.814	0.689	12.5	0.606	0.619	1.3
AP	0.788	0.723	6.5	0.641	0.645	0.4
PP	0.684	0.672	1.2	0.607	0.648	4.1
DP	0.667	0.743	7.6	0.59	0.652	6.2
CEUS	0.735	0.798	6.3	0.653	0.67	1.7
B + CEUS	**0.818**	**0.802**	**1.6**	**0.667**	**0.688**	**2.1**

Each DL model was built on the corresponding modality; the models were trained on data from one and validated in the other across two centers. Cross-center robustness was assessed by the Delong test between two centers within internal and external datasets (*P >* 0.05, for all modalities). ΔAUC = |AUC Center1 – AUC Center2|. DL: Deep learning, B: B mode, AP: Arterial phase, PP: Portal phase, DP: Delayed phase, CEUS: Contrast-enhanced ultrasound

For radiomics models, we built two series of models, one with a ROI segmented on the visible broadline of the lesions and the other with n ROI enlarged by 20% around the initial segmentation. The radiomics models showed improved performance with enlarged ROIs in most modalities, and statistical improvement was achieved in CEUS and B + CEUS modalities using the Delong test (*P* < 0.05). The enlarged-ROI radiomics models provided the best AUC of 0.87 in the B + CEUS model during internal validation, whereas the same modality trained in the other center yielded a relatively lower AUC of 0.749, a 12% drop-off for the optimal modality, showing relatively limited robustness in the radiomics model; however, the Delong test did not show a difference in the cross-institutional comparison (*P >* 0.05, for all modalities). The performance of the radiomics models is presented in Table 3. Both DL and radiomics models were limitedly generalizable.

**Table 3:** Evaluation of radiomics performance on MVI prediction using reciprocal cross-institutional validation of multidata models within original and enlarged ROIs

Radiomics model AUCs	Validation sets		ΔAUC (%)	External sets		ΔAUC (%)
Modality	Center 1	Center 2		Center 1	Center 2	
B	0.656	0.589	6.7	0.657	0.636	2.1
AP	0.615	0.603	1.2	0.682	0.651	3.1
PP	0.684	0.686	0.2	0.691	0.668	2.3
DP	0.717	0.717	0	0.7	0.667	3.3
CEUS	0.719	0.762	4.3	0.71	0.607	10.3
B + CEUS	0.703	0.682	2.1	0.679	0.601	7.8
B ROI-enlarged	0.676	0.621	5.5	0.682	0.635	4.7
AP ROI-enlarged	0.695	0.659	3.6	0.708	0.676	3.2
PP ROI-enlarged	0.689	0.682	0.7	0.681	0.649	3.2
DP ROI-enlarged	0.723	0.738	1.5	0.696	0.664	3.2
CEUS ROI-enlarged	0.865* ^*P* = 0.036^	0.756	10.9	0.718	0.685* ^*P* = 0.017^	3.3
B + CEUS ROI-enlarged	0.869* ^*P* = 0.033^	**0.749**	**12**	**0.697**	**0.646**	**5.1**

Each radiomics model was built on the corresponding modality, with original and enlarged ROIs; the models were trained on data from one and validated in the other across two centers. The Delong test was performed between original and enlarged ROIs within the same modalities (“*” indicates a significant difference), and performance between the two centers (*P >* 0.05, for all modalities). ΔAUC = |AUC Center1 – AUC Center2|. B: B mode, AP: Arterial phase, PP: Portal phase, DP: Delayed phase, CEUS: Contrast-enhanced ultrasound, ROI: Region of interest

Optimal model in DL and radiomics models with clinical information.

B + CEUS was selected as the optimal model construction modality. Specifically, for radiomics models, ROI-enhanced segmentation enhanced the B + CEUS model. Additionally, the clinical data of AFP were further incorporated into the models. As shown in Table 4, combining the optimal modality with AFP statistically boosted the DL models in the external sets (Delong test, *P* < 0.05), while adding AFP provided no gains for radiomics and appeared to reduce its accuracy overall. The ΔAUC (Delta AUC) in these tables represents the difference in AUC values between the two centers and can be used as an indicator of model robustness. The lower ΔAUC value of the optimal DL model (ΔAUC: 1.6–2.1% vs. 5.2–12%, DL vs. Radiomics) suggests that the DL model exhibits good robustness across centers, which is maintained even when clinical factors (AFP) are added to the DL model (ΔAUC: 0.8–2.8%).

**Table 4:** Comparison of the optimal DL and radiomics models for MVI prediction combined with clinical factors

Method AUCs	Modality	Validation sets		ΔAUC (%)	External sets		ΔAUC (%)
		Center 1	Center 2		Center 1	Center 2	
DL	B + CEUS	0.818	0.802	1.6	0.667	0.688	2.1
	B + CEUS + AFP	**0.818**	**0.81**	**0.8**	0.71* ^*P* = 0.025^	0.738* ^*P* = 0.036^	**2.8**
Radiomics	B + CEUS ROI-enlarged	0.869	0.749	12	0.697	0.646	5.2
	B + CEUS ROI-enlarged + AFP	0.823	0.699	12.4	0.669	0.636	3.3

The optimal models in DL and radiomics were compared to the ones combining clinical factor alpha-fetoprotein (AFP). The Delong test was performed between combined AFP models vs. optimal models, “*” indicates significant difference. ΔAUC = |AUC Center1 – AUC Center2|. AFP: Alpha-fetoprotein

To provide context, we analyzed physicians’ MVI detection capabilities when relying on factors such as tumor size, margin characteristics, capsule completeness, and portal vein invasion. The results are compared to the optimal models in Table 5. Figure 3 displays histograms of the AUC comparison of DL and radiomics models in CEUS and B+CEUS modalities and ROC curves comparing the optimal models with the doctor. 

**Table 5:** External validation performance of doctor, the optimal DL model combined with AFP and the optimal radiomics model for MVI prediction

Modality	AUC	ACC	SEN	SPE	PPV	NPV
Doctor	0.603	0.604	0.598	0.608	0.711	0.483
The optimal DL + AFP	**0.738**	**0.710**	**0.606**	**0.765**	**0.577**	**0.786**
The optimal Ra	0.697	0.674	0.619	0.714	0.613	0.719

DL: Deep learning, Ra: Radiomics, ACC: Accuracy, AUC: Area under receiver operation curve, SEN: Sensitive, SPE: Specificity, PPV: Positive predictive value, NPV: Negative predictive value

## Discussion

In this study, we compared DL and radiomics techniques for preoperative prediction of MVI in patients with HCC. Through rigorous cross-validation and external testing, DL models optimized for B + CEUS images demonstrated superior diagnostic accuracy. Incorporating AFP levels, DL models achieved AUCs of 0.818 and 0.738 on the internal and external datasets, respectively. In contrast, during internal validation, despite higher internal validation AUCs of up to 0.869 using enlarged ROI segmentation, the radiomics models showed a considerable drop to 0.749 in cross-institutional internal data. This indicates susceptibility to overfitting and raises questions about the robustness of radiomics methodology. The innovative aspect of our study was the cross-institutional validation of the DL and radiomics models applied to ultrasound images for predicting MVI in HCC.

In this study, we chose CEUS as the main imaging modality rather than MRI or CT, mainly based on the following considerations: First, CEUS has advantages such as real-time imaging, non-radiation, and lower cost, which are more suitable for widespread clinical application. Second, CEUS has a higher sensitivity for microvascular imaging than CT and MRI, which has potential advantages in detecting MVI. The results of this study showed that the DL model combining B-mode CT and CEUS achieved AUCs of 0.818 and 0.738 in internal validation and external testing, respectively, which is comparable to previous studies based on CT/MRI. For example, the DL model based on CT imaging by Liu et al. achieved AUCs of 0.845 and 0.777 in internal validation and external testing, respectively. This indicates that DL model based on CEUS is likely to be comparable to that of CT/MRI for MVI prediction.

However, as a real-time and operator-dependent technique, CEUS faces challenges in standardized imaging, which differs from the standardized imaging protocols of MRI and CT. To achieve model performance comparable to that of MRI and CT-based methods, it is necessary to address the standardization issues in CEUS imaging. We found that the DL approach has advantages in addressing the drawbacks of non-standardized CEUS imaging. By comparing the performance of DL and radiomics models, we found that the DL models demonstrated better robustness across institutions, whereas the radiomics models were more prone to overfitting and had limited robustness across institutions. This finding suggests that the DL approach can improve the clinical application of CEUS for MVI prediction and provide a possible solution to overcome the challenges associated with CEUS standardization.

MVI status is a crucial factor for clinicians in assessing patient prognosis. This parameter impacts postoperative relapse and survival rates in resectable cases [17]. Most existing studies based on DL or radiomics demonstrated high prediction performance for MVI, with validation AUCs reaching 0.744–0.947. However, the majority of the participants employed internal validation on samples from the same institution as the training cohort. Only a few studies have examined generalizability through external validation across institutions, where performance dropped substantially (e.g., Liu et al. internal 0.845 vs. external AUC 0.777) [18, 19]. This highlights a major limitation, in that high reported accuracy may not translate to new patient populations in real-world clinical practice. Thus, there is a need for more rigorous evaluation of model robustness and stability across diverse datasets. Additionally, direct comparisons between radiomics and DL are limited. Jiang et al. conducted one of the first head-to-head comparisons by building separate radiomics and DL models on the same CT data. Their DL model achieved a slightly higher accuracy than that of radiomics for MVI prediction (AUC: 0.906 vs. 0.887) although both models outperformed subjective evaluations. Our study represents one of the first efforts in ultrasound imaging to directly compare radiomics and DL models for MVI prediction using both internal and external multi-center validation. This approach allows for rigorous benchmarking of the real-world generalizability and clinical utility of the two approaches.

The study’s retrospective design may carry inherent biases, including selection bias, which could impact the models’ predictive capabilities. Moreover, in terms of data selection, using a single 2D slice results in loss of volumetric data, as is the case for the three still images from contrast ultrasound resulting in loss of dynamic enhancement information on blood flow. Furthermore, although the sample size is relatively large sample and the study is conducted across multiple centers, a larger sample size is required to optimize the models. Moreover, although we compared the generalizability of both the DL and radiomics models, which showed that the latter had better performance, further improvements in generalizability are still required before clinical use.

In conclusion, our findings demonstrated the potential of DL models using combined B-mode and CEUS imaging to predict MVI in patients with HCC. Through rigorous internal and external validation, the DL models showed superior generalizability compared to the radiomics models, which suffered considerable performance reductions on external data despite high internal accuracy, as well as diversity on the in/external sets AUC across centers. This indicates that the DL approach may be more robust and clinically applicable across diverse institutions. Multicenter validation represents an important advance in realistically benchmarking model performance. Overall, this study provides valuable insights into the comparative effectiveness of DL and radiomics for preoperative MVI prediction using ultrasound imaging. DL modeling shows promise, but further optimization and validation are necessary to translate high accuracy into clinical utility for improving prognostic assessment and surgical planning for patients with HCC.

## Data Availability

No datasets were generated or analysed during the current study.

## Abbreviations

ΔAUC: Delta AUC
AFP: Alpha-fetoprotein
AUC: Area under the curve
B: B mode
CEUS: Contrast-enhanced ultrasound
DKI: Diffusion kurtosis imaging
DL: Deep learning
DP: Delayed phase
GS: Scheuer GS classification
HBV: Hepatitis B virus
HCC: Hepatocellular carcinoma
HCV: Hepatitis C virus
LASSO: Least Absolute Shrinkage and Selection Operator
MVI: Microvascular invasion
NPV: Negative predictive value
PP: Portal phase
PPV: Positive predictive value
RFA: Radiofrequency ablation
ROC: Receiver operating characteristic
ROI: Region of interest
S: Stage of fibrosis
SEER: Surveillance, Epidemiology, and End Results
SEN: Sensitivity
TACE: Transcatheter arterial chemoembolization
TIC: Time-intensity curve
